# Role of Synaptic Inhibition in the Coupling of the Respiratory Rhythms that Underlie Eupnea and Sigh Behaviors

**DOI:** 10.1523/ENEURO.0302-19.2020

**Published:** 2020-06-05

**Authors:** Daniel S. Borrus, Cameron J. Grover, Gregory D. Conradi Smith, Christopher A. Del Negro

**Affiliations:** Department of Applied Science, Integrated Science Center, William & Mary, Williamsburg, VA 23185

**Keywords:** breathing, central pattern generator, preBötzinger complex

## Abstract

The preBötzinger complex (preBötC) gives rise to two types of breathing behavior under normal physiological conditions: eupnea and sighing. Here, we examine the neural mechanisms that couple their underlying rhythms. We measured breathing in awake intact adult mice and recorded inspiratory rhythms from the preBötC in neonatal mouse brainstem slice preparations. We show previously undocumented variability in the temporal relationship between sigh breaths or bursts and their preceding eupneic breaths or inspiratory bursts. Investigating the synaptic mechanisms for this variability *in vitro*, we further show that pharmacological blockade of chloride-mediated synaptic inhibition strengthens inspiratory-to-sigh temporal coupling. These findings contrast with previous literature, which suggested glycinergic inhibition linked sigh bursts to their preceding inspiratory bursts with minimal time intervals. Furthermore, we verify that pharmacological disinhibition did not alter the duration of the prolonged interval that follows a sigh burst before resumption of the inspiratory rhythm. These results demonstrate that synaptic inhibition does not enhance coupling between sighs and preceding inspiratory events or contribute to post-sigh apneas. Instead, we conclude that excitatory synaptic mechanisms coordinate inspiratory (eupnea) and sigh rhythms.

## Significance Statement

Normal breathing consists of eupnea and sigh breaths, which differ in their magnitude, frequency, and function. Both breath types emerge from a brainstem microcircuit that coordinates their timing. Here, we advance understanding of these rhythms by assessing the nature and strength of their coordination, and by showing that synaptic inhibition does not constrain their temporal coupling in contrast to conventional understanding. This study elucidates synaptic mechanisms linking oscillations of different amplitude and frequency within one core oscillator microcircuit.

## Introduction

Under physiological conditions breathing behavior consists of two interleaving rhythms and motor patterns: eupnea and sighing. Eupnea is the normal unlabored breathing that underlies rhythmic lung ventilation and drives alveolar gas exchange. Eupnea occurs at ∼1–4 Hz in rodents (0.2–0.3 Hz in humans); each breath ventilates a small fraction of lung capacity. Sighs are also inspiratory breaths, but the volume of inhaled air during a sigh is two to five times that of a normal breath, and sigh frequency is an order of magnitude lower than the eupnea rhythm (Li and Yackle, 2017). Sighs reinflate collapsed (or collapsing) alveoli and are essential for optimal pulmonary function. Typically, sighs appear to ride atop ongoing eupneic breaths (Cherniack et al., 1981; Orem and Trotter, 1993), which suggests that periodically, but at a much lower frequency, the eupnea cycle triggers the sigh. After a sigh, the next eupneic breath is delayed for a duration roughly equivalent to one additional eupneic cycle (Cherniack et al., 1981; Orem and Trotter, 1993). This delay, which we refer to as the post-sigh apnea, suggests that sighs prolong the time to the next eupneic breath. Eupnea and sigh breathing rhythms thus appear to be coupled, most likely via neural microcircuits of the brainstem that generate and control breathing movements.

In mammals, eupnea and sigh rhythms emanate from the preBötzinger complex (preBötC) of the lower brainstem (Smith et al., 1991; Del Negro et al., 2018). Both rhythms are maintained in reduced slice preparations that isolate the preBötC as well as inspiratory premotor and motor neurons, and thus encapsulate a minimal breathing-related model system (Lieske et al., 2000; Ruangkittisakul et al., 2008; Chapuis et al., 2014). Because eupnea refers to behavior in living animals, inspiratory is the appropriate nomenclature for eupnea-related activity in slice preparations. Inspiratory rhythm depends on network properties, in which recurrent excitation among glutamatergic interneurons is rhythmogenic (Funk et al., 1993; Rekling et al., 2000; Del Negro et al., 2002; Wallén-Mackenzie et al., 2006; Feldman and Kam, 2015; Ashhad and Feldman, 2020). The rhythmogenic mechanism of sighs is unknown, but maintenance of the sigh rhythm depends on neuropeptides released by parafacial respiratory interneurons (Li et al., 2016) as well as excitatory ionotropic and metabotropic receptor-mediated synaptic transmission (Lieske and Ramirez, 2006a,b).

Inspiratory bursts appear to trigger sigh-related bursts, and in turn, sigh-related bursts delay the next inspiratory burst by almost an entire cycle (Lieske et al., 2000; Tryba et al., 2008). These observations *in vitro* mirror the *in vivo* coupling behavior described above, which suggest that the mechanisms coupling inspiratory and sigh rhythms are contained within the preBötC and can be examined at the cellular and synaptic level *in vitro*.

What mechanisms couple these two rhythms? The only existing data suggest that glycinergic synaptic inhibition links the sigh-related burst to its preceding inspiratory burst, thus giving rise to the biphasic shape in which the inspiratory burst appears to trigger the sigh (Lieske et al., 2000). A recent mathematical model (Toporikova et al., 2015) posits two discrete systems for generating eupnea and sigh oscillations. The model inspiratory system acts on the sigh system via synaptic inhibition such that sigh bursts emerge via an escape-like process triggered by disinhibition at the tail end of inspiratory bursts. The model also suggests that the sigh system projects to the inspiratory system via fast excitatory synapses, and the strength of its excitation leads to a transient state of refractoriness, i.e., the post-sigh apnea, in the coupled system. However, the post-sigh apnea might also be attributable to synaptic inhibition from the sigh system onto the inspiratory rhythm generator.

Here, we challenge two longstanding ideas regarding the sigh rhythm: that when a sigh occurs, it emerges immediately following the associated eupneic breath, and that synaptic inhibition is responsible for this temporal relationship. First, we describe previously undocumented variability in the timing of a sigh breath relative to the previous eupneic event. We show, both *in vivo* and *in vitro*, that a sigh event can occur simultaneously, and even precede, the inspiratory event. Next, we block glycinergic transmission and show that disinhibiting the preBötC *in vitro* does not uncouple the eupnea- and sigh-related rhythms, but in fact appears to couple them more strongly. We obtain similar results when simultaneously blocking GABAergic and glycinergic transmission.

We extend our investigation of synaptic inhibition in coupling the two breathing rhythms by analyzing the post-sigh apnea *in vitro* before and after blockade of ionotropic inhibition. We show the duration of the post-sigh apnea does not depend on glycinergic or GABAergic transmission and we infer that the post-sigh apnea reflects a refractory state attributable to postsynaptic membrane properties evoked by the sigh burst. Lastly, we measure the chloride reversal potential (E_Cl_) in putatively rhythmogenic preBötC neurons and meta-analyze the development of E_Cl_ in mice and rats, to verify that glycine and GABA_A_ synapses are inhibitory postnatally, and not functionally excitatory as they are during embryonic development (Ren and Greer, 2006; Delpy et al., 2008). We propose that the eupnea and sigh rhythms are coupled predominantly by excitatory (rather than inhibitory) synaptic mechanisms.

## Materials and Methods

### Ethical approval and animal use

The Institutional Animal Care and Use Committee at our institution approved these protocols, which conform to the policies of the Office of Laboratory Animal Welfare (National Institutes of Health) and the guidelines of the National Research Council [National Research Council (U.S.), 2011]. CD-1 mice (Charles River) and genetically modified mice (described below) were maintained on a 14/10 h light/dark cycle at 23°C and were fed *ad libitum* with free access to water.

To obtain patch-clamp recordings from preBötC neurons derived from progenitors that express the embryonic transcription factor Developing brain homeobox 1 (i.e., *Dbx1*), we used two Cre-driver mouse strains: homozygous knock-in mice generated by inserting an *IRES-CRE-pGK-Hygro* cassette in the 3′ untranslated region (UTR) of the *Dbx1* gene, i.e., *Dbx1^Cre^* mice (Bielle et al., 2005; IMSR catalog #EM:01924, RRID:IMSR_EM:01924) and homozygous knock-in mice generated by inserting an IRES-CreER^T2^ cassette in the 3′ UTR of the *Dbx1* gene, which provides conditional Cre recombinase expression following activation of the tamoxifen-sensitive estrogen receptor, i.e., *Dbx1^CreERT2^* mice (Hirata et al., 2009; Picardo et al., 2013; IMSR catalog #JAX:028131, RRID:IMSR_JAX:028131).

We crossed females of both strains with males from a reporter strain featuring Cre-dependent expression of the fluorescent Ca^2+^ indicator GCaMP6f dubbed Ai148 by the Allen Institute (Daigle et al., 2018; IMSR catalog #JAX:030328, RRID:IMSR_JAX:030328). We refer to their offspring as Dbx1;Ai148 mice. During neonatal development and through adulthood, Dbx1;Ai148 mice express GCaMP6f in *Dbx1*-derived cells, the majority of which are neurons (Kottick et al., 2017).

### Breathing-related measurements *in vitro*

Mouse pups of both sexes were anesthetized by hypothermia and then killed by thoracic transection at postnatal day (P)0–P4. The neuraxis was removed in <2 min and further dissected in artificial CSF (aCSF) containing the following: 124 mm NaCl, 3 mm KCl, 1.5 mm CaCl_2_, 1 mm MgSO_4_, 25 mm NaHCO_3_, 0.5 mm NaH_2_PO_4_, and 30 mm dextrose equilibrated with 95% O_2_-5% CO_2_, pH 7.4. Isolated neuraxes were glued to an agar block and then cut in the transverse plane to obtain a single 550-μm-thick slice that exposed the preBötC at its rostral face. Atlases for wild-type and *Dbx1* reporter mice show that the loop of the inferior olive and the semicompact division of the nucleus ambiguus (NAsc) collocate with the preBötC during early postnatal development (Ruangkittisakul et al., 2011, 2014). Slices were then perfused with aCSF at 28°C in a recording chamber either below a stereomicroscope that enabled us to position suction electrodes under visual control, or on a fixed-stage microscope with dipping objectives and epifluorescence illumination to visually identify GCaMP6f-expressing (rhythmically active) *Dbx1*-derived neurons for patch-clamp recordings.

In both configurations, we elevated extracellular K^+^ concentration ([K^+^]_o_) to 9 mm to increase preBötC excitability (Funk and Greer, 2013). Inspiratory-related motor output was recorded from the hypoglossal (XII) nerve rootlets, which are captured in transverse slices along with the XII motoneurons and their axon projections to the nerve rootlets, using suction electrodes and a differential amplifier. Field potentials were recorded from the preBötC by forming a seal over it with a suction electrode at the slice surface. Amplifier gain was set at 2000 and the bandpass filter was set at 300–1000 Hz. XII and preBötC bursts were full wave rectified and smoothed for display and quantitative analyses of burst events.

We obtained on-cell patch-clamp recordings under visual control. Patch pipettes were fabricated from borosilicate glass (OD: 1.5 mm, ID: 0.86 mm, 4–6 MΩ in bath) and filled with solution containing 150 mm KCl and 10 mm HEPES). We added gramicidin (CAS number 1405-97-6, product G0550000 from Millipore Sigma) acutely at the start of the experiment from stock solution (2-mg gramicidin per 1-ml dimethyl sulfoxide) such that the final concentration was 20 μg/ml. We also added Alexa Fluor 568 hydrazide (50 μm, ThermoFisher Scientific) to the patch solution, which verifies the integrity of the gramicidin perforated patch as GCaMP6f fluorophore is expressed in the soma, whereas Alexa Fluor 568 is limited to the adjacent pipette. Patch pipettes with a bath resistance of 4–6 MΩ were backfilled first with a drop of gramicidin-free patch solution to ensure gramicidin molecules did not interfere with the initial seal to the membrane. After backfilling, 200 μl of patch solution with gramicidin was added to the pipette. We employed an EPC-10 patch-clamp amplifier exclusively in current-clamp mode (HEKA Instruments) because perforated patches cause a large access resistance at the interface between pipette and target neuron, which produces a voltage-divider circuit and precludes accurate voltage clamp.

We acquired and digitized the signals at 4 kHz with a low-pass filter set to 1 kHz using a 16-bit analog-to-digital converter (AD Instruments). All recordings were corrected offline for a liquid junction of 3.74 mV (Barry and Lynch, 1991; Neher, 1992).

For both field recordings and associated perforated-patch experiments, the glycine receptor antagonist strychnine hydrochloride (CAS number 1421-86-9, product S8753, Millipore Sigma) and the GABA_A_ receptor antagonist picrotoxin (CAS number 124-87-8, product P1675, Millipore Sigma) were bath-applied at 5 μm.

All gramicidin perforated-patch experiments began no earlier than 30 min after achieving a seal on the plasma membrane exceeding 1 GΩ (i.e., gigaohm seal), which was sufficient for gramicidin to form ionophores and thus allow intracellular access and current-clamp recording. After we observed a stable recording configuration for 10 min, which we considered the control conditions for subsequent analysis, then we applied strychnine and picrotoxin.

When locally applying GABA_A_ and glycine receptor agonists (muscimol and glycine) onto patch-recorded neurons, we fabricated similar pipettes (as described above) from which to eject glycine and muscimol (dubbed “puffer” pipettes). Puffer pipettes were filled with 150 μm glycine (CAS number 56-40-6, Millipore Sigma) and 30 μm muscimol (CAS number 2763-96-4, Millipore Sigma) diluted into aCSF containing 9 mm [K^+^]_o_ (as described above). Thirty minutes after forming a gigaohm seal on the plasma membrane, the puffer pipette was positioned to within ∼50 μm of the neuron being recorded. Glycine and muscimol were ejected using 7–9 psi pressure pulses lasting 25–200 ms, which we triggered by TTL commands from the EPC-10 amplifier.

Patch-recorded preBötC neurons were identified as neurons by their ability to discharge action potentials, recognizable ∼30 min after forming a gigaohm seal. The high impedance of gramicidin patches filters high frequency signals like action potentials, thus spike amplitude peaks below –10 mV while the integrity of the patch is maintained. The developmental genetic origin of neurons patched from wild-type preparations was not specified, thus we refer to these neurons as unspecified. Neurons patched in Dbx1;Ai148 mouse slices, which were rhythmically active in sync with XII motor output, are referred to as Dbx1 neurons. For puffing experiments, we added 1 μm tetrodotoxin (TTX) to the bath after >30 min to prevent spike-driven chemical synaptic transmission and isolate postsynaptic responses to muscimol and glycine. We held preBötC neurons at a range of baseline membrane potentials using bias currents. We measured membrane potential trajectories in response to puffed glycine and muscimol, in which the previous 2 s of membrane voltage were used as baseline.

### Breathing-related measurements *in vivo*

We measured breathing in unrestrained, adult mice of both sexes (*n* = 7 mice total), using a whole-body plethysmograph (EMKA Technologies) with a balanced flow rate of 1 l/min in normoxia (21% O_2_ and 79% N_2_). The mice were placed in the sealed chamber with constant air flow 10 min before data collection during each session for acclimation. We observed the mice during every session. Locomotion (exploring), grooming, and sniffing (with synchronized whisking) entrain and modify breathing so we only analyzed epochs of calm breathing absent orofacial or motor behaviors. A pneumotachograph with access to the chamber containing the mouse, and a reference chamber open to room air, were included in the circuit and connected to a differential pressure transducer (AD Instruments, RRID:SCR_017551). The raw data reflect airflow, which was acquired and analyzed in LabChart 8 software (AD Instruments). Airflow was digitally integrated to determine volume changes during breathing and in particular to determine tidal volume (V_T_). The pressure transducer was calibrated by injecting 0.2 ml of air into the chamber during each trial. We analyzed continuous 60-min sessions of plethysmography. Epochs of calm breathing contain eupnea and sighs. Sighs were distinguished by inhaled volume exceeding V_T_ by 2–3× (the inspired air during a sigh draws on the inspiratory reserve volume of the lungs, and thus exceeds V_T_ by definition) and by the presence of a post-sigh apnea, i.e., a pause in breathing 1.3× longer than the average interbreath interval during eupnea.

### Identification and analysis of sigh bursts

**Figure 1.**
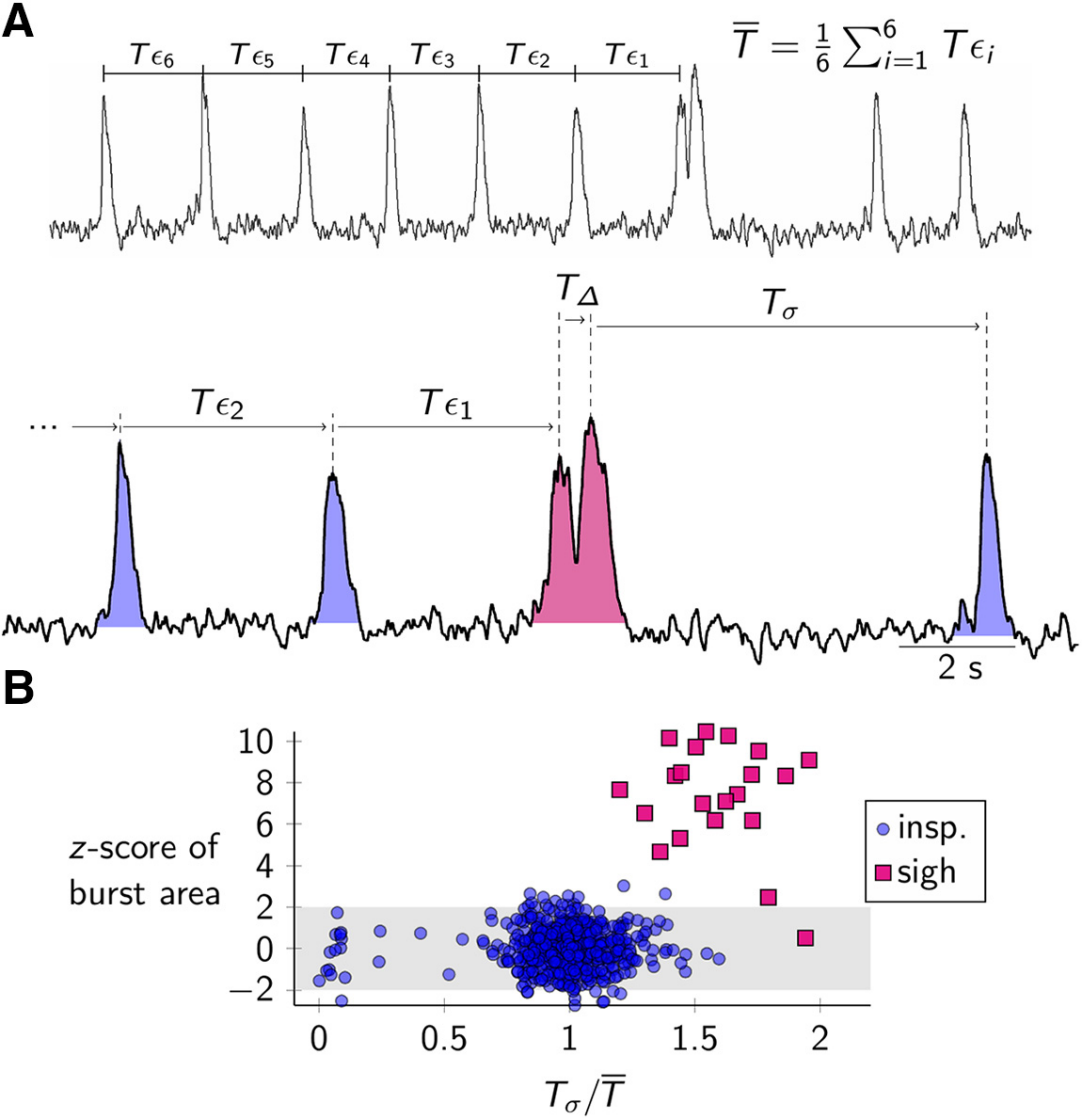
***A***, Field recording of preBötC activity, which includes inspiratory and sigh bursts. Average inspiratory cycle time ($\bar{T}$) is calculated from six preceding cycle periods ($T_{\epsilon_{1}}...T_{\epsilon_{6}}$). $T_{\epsilon }$ represents period of a single inspiratory cycle; $T_{\Delta }$ represents the inspiratory-sigh interval; $T_{\sigma }$ reflects the duration of the post-sigh apnea. ***B***, A scatter plot showing *z* score transformation of burst area plotted against the post-sigh apnea duration, $T_{\sigma }$ (normalized to the average inspiratory cycle time, $\bar{T}$); *z* score distribution is calculated from all bursts from this slice.

We measured burst area and cycle time for each event in preBötC field recordings (Fig. 1). We distinguished a sigh burst from an inspiratory burst by considering the area of an event in conjunction with the cycle time following that event. Sigh bursts have *z* scores (mean/SD) for burst area that almost always exceed 2 (and are most often in the range 4–10), whereas inspiratory bursts have an average *z* score of 0 (range –2–2). Sigh bursts are followed by a cycle time as long or longer than 1.3× the average cycle time for all events lumped together. Figure 1*A*,*B* illustrates these measurements for a typical field recording.

The average inspiratory cycle time ($\bar{T}$) is computed from the six inspiratory cycles preceding the sigh ($T_{\epsilon_{1}}...T_{\epsilon_{6}}$). $T_{\Delta }$ is the inspiratory-sigh interval computed as the peak time of the sigh burst minus the peak time of the inspiratory burst. $T_{\sigma }$ is the duration of the post-sigh apnea. We normalize $T_{\Delta }$ and $T_{\sigma }$ by $\bar{T}$ to minimize the slice-by-slice variation in frequencies.

### Measurements and statistics

We analyzed data and computed statistics using LabChart 7 & 8 (AD Instruments), MATLAB 2018b (MathWorks, RRID:SCR_001622), and Igor Pro 8 (Wavemetrics, RRID:SCR_000325). We describe the statistical hypothesis tests used as they appear in the main text (Table 1).

**Table 1:** Summary of statistics from figures

	Figure	Data structure	Type of test	Power
a	4	Undefined	Kolmogorov–Smirnov test	*p* = 0.011
b	4	Undefined	Kolmogorov–Smirnov test	*p* = 4.0E-12
c	5	Normally Distributed	One-way ANOVA	*p* = 0.14
d	6*F*	Normally Distributed	Paired *t* test	*p* = 0.0368
e	6*H*,*I*	Undefined	Linear regression	E_Cl_ = –47 mV *r*^2^ = 0.97
f	6*I*	Undefined	Linear regression	E_Cl_ = –46 mV *r*^2^ = 0.94
g	6*I*	Undefined	Linear regression	E_Cl_ = –43 mV *r*^2^ = 0.91
h	6*I*	Undefined	Linear regression	E_Cl_ = –45 mV *r*^2^ = 0.85
i	6*I*	Undefined	Linear regression	E_Cl_ = –65 mV *r*^2^ = 0.95
j	6*I*	Undefined	Linear regression	E_Cl_ = –45 mV *r*^2^ = 0.87
k	6*I*	Undefined	Linear regression	E_Cl_ = –58 mV *r*^2^ = 0.90
l	6*I*	Undefined	Linear regression	E_Cl_ = –44.21 mV *r*^2^ = 0.94
m	6*I*	Undefined	Linear regression	E_Cl_ = –45.74 mV *r*^2^ = 0.28
n	6*I*	Undefined	Linear regression	E_Cl_ = –45.98 mV *r*^2^ = 0.94
o	6*I*	Undefined	Linear regression	E_Cl_ = –48.93 mV *r*^2^ = 0.85

## Results

### Sigh breaths follow eupneic breaths with variable time intervals

**Figure 2.**
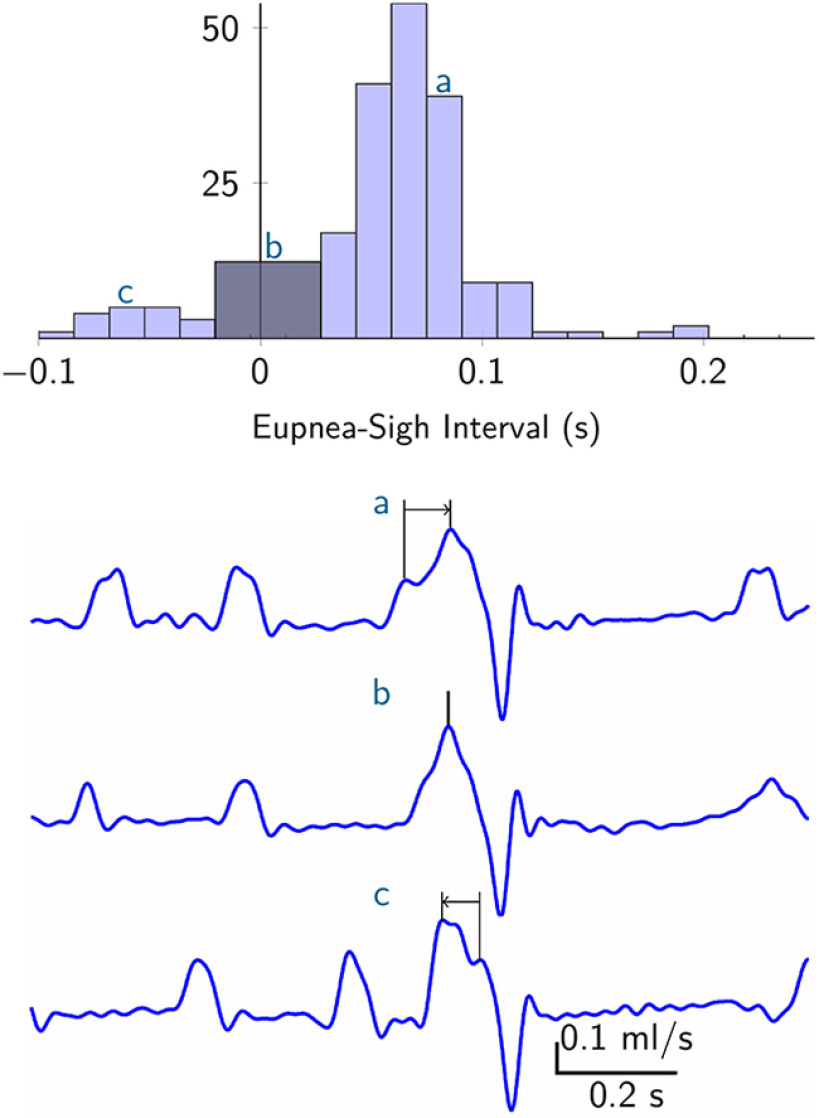
Variable timing of sigh breaths *in vivo*. Top, Histogram of the eupnea-sigh time intervals. Bin size is 0.016 s. Intervals near zero were pooled into one bin of size 0.048 s because distinct eupnea-related and sigh-related peaks in the signal could not be distinguished. Bottom, Sample sweeps from plethysmography recordings from one mouse. Lower case letters a, b, and c illustrate eupnea-sigh intervals that appear in corresponding bins plotted in the histogram above.

We recorded breathing in unanesthetized adult mice, analyzing 229 sigh breaths across seven animals. We measured the time interval between a sigh and its associated (generally preceding) eupneic breath, which we refer to as the eupnea-sigh interval. Figure 2 shows the distribution of the eupnea-sigh intervals with three representative traces corresponding to eupnea-sigh intervals that fall within three characteristic parts of the distribution. In 174 of the 229 sighs (76%), the sigh emerged after the eupneic breath, leading to a positive eupnea-sigh interval (Fig. 2, trace a). This represents the canonical sigh pattern, whereby the inspired volume of the sigh builds off, and exceeds, the tidal volume marking the peak of a eupneic breath. Surprisingly however, the remaining 55 sighs (24%) did not follow the canonical pattern. In 37 cases (16%), the sigh appeared unaccompanied by a eupneic breath. We infer that the eupneic and sigh event occurred simultaneously, or close enough together so as to be indistinguishable, thus leading to an observed event interval of 0 s (Fig. 2, trace b). In 18 cases (8%), the sigh breath was followed by a eupneic breath whose peak corresponds to tidal volume, then followed by a post-sigh apnea. This type of sigh pattern led to a negative eupnea-sigh interval (Fig. 2, trace c). The presence of null and negative eupnea-sigh intervals (Fig. 2, traces b and c, respectively) suggest that eupneic breaths are not obligatory for triggering a sigh.

### Sigh bursts follow inspiratory bursts with variable time intervals

**Figure 3.**
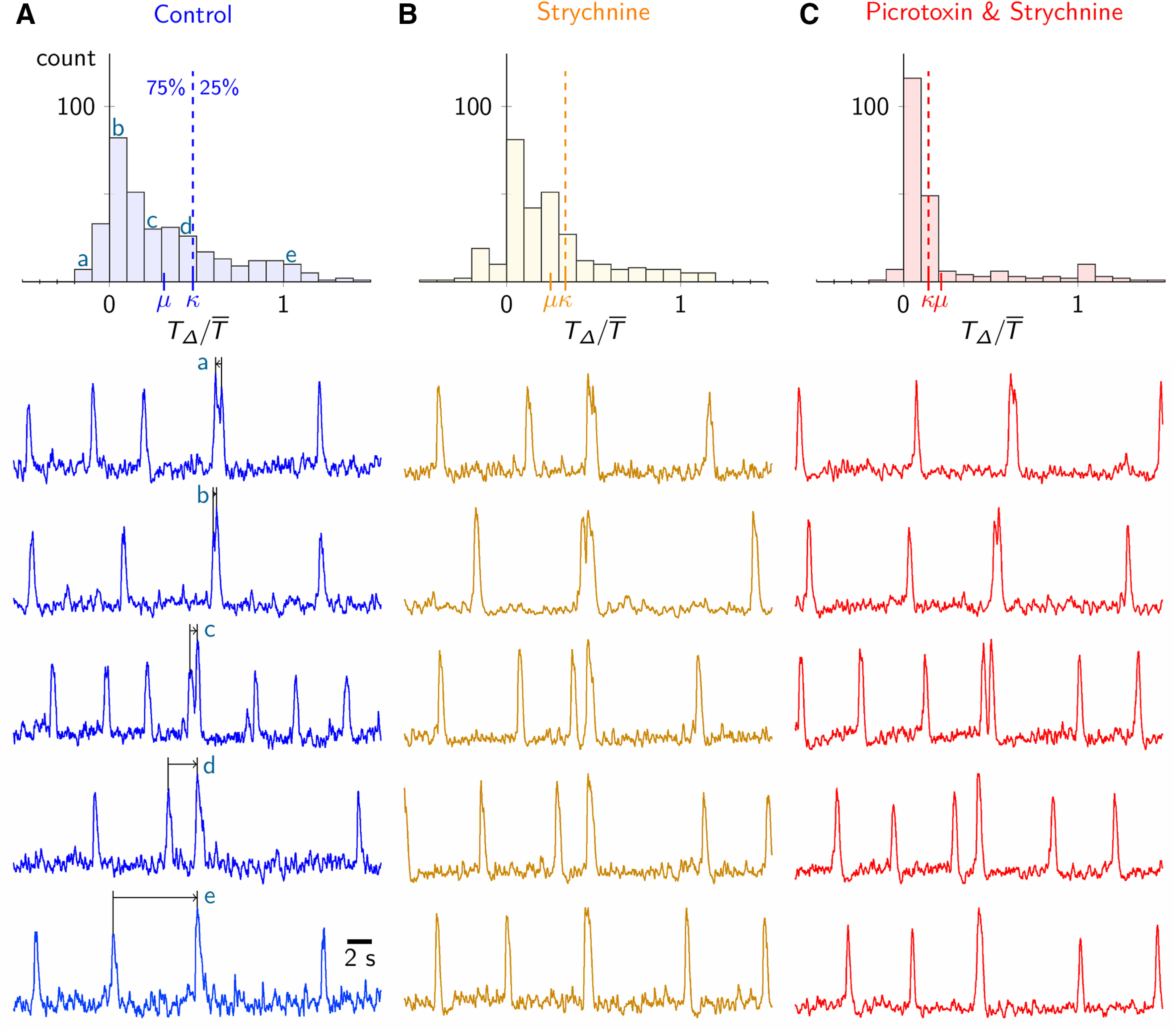
Variable timing of sigh bursts *in vitro*. ***A***, Histogram of normalized inspiratory-sigh intervals ($T_{\Delta }/\bar{T}$) for all sigh bursts in all 13 rhythmically active slices under control conditions. The width of each bar is $0.1T_{\Delta }/\bar{T}$; μ marks the mean of the distribution, and κ denotes the 75th percentile line. Below are representative preBötC field recording traces depicting a variety of inspiratory-sigh intervals. Traces come from different slices. Lower case letters a, b, c, d, and e connect representative traces with their position on the inspiratory-sigh interval histogram above. ***B***, ***C***, Identical inspiratory-sigh interval analysis in the presence of 5 μm strychnine (***B***) as well as both 5 μm strychnine and 5 μm picrotoxin (***C***).

We next investigated the coupling relationship between eupnea and sigh rhythms using slice preparations that retain the preBötC and spontaneously generate inspiratory and sigh-related activity measurable in the preBötC and XII nerve rootlets. We measured 343 sigh bursts in 13 slice preparations (26 ± 7 sigh bursts per slice) via a field recording electrode. We measured the time interval between a sigh burst and its associated inspiratory burst, which we refer to as the inspiratory-sigh interval (Fig. 3*A*). To account for the intrinsic slice-to-slice variation in inspiratory frequency, we normalized inspiratory-sigh interval ($T_{\Delta }$) by the average of the six preceding inspiratory cycle periods ($\bar{T}$). Figure 3*A* shows the distribution of inspiratory-sigh intervals (top), as well as representative field recordings of sigh bursts with different inspiratory-sigh intervals that map to specific parts of the distribution (Fig. 3*A* shows the correspondence via lower case letters a–e).

Inspiratory and sigh events recorded from XII motor output closely resemble the integrated preBötC field recording when considering inspiratory-sigh timing. We analyze the integrated field recording exclusively because it reflects activity of the core rhythmogenic microcircuits in the preBötC.

Inspiratory bursts tend to precede sigh bursts, as the distribution of inspiratory-sigh intervals is weighted toward positive values of $T_{\Delta }/\bar{T}$ close to 0 (Fig. 3*A*; e.g., trace b). However, inspiratory-sigh intervals varied considerably. For example, only 133 sighs (39%) appeared within the first fifth of the inspiratory cycle. Rather, we observed inspiratory-sigh intervals that ranged across the entire inspiratory cycle (Fig. 3*A*, traces b–e). Additionally, we observed 40 sigh bursts (12%) that appeared to be followed by inspiratory bursts and then post-sigh apneas, thus indicating a negative inspiratory-sigh interval (Fig. 3*A*, trace a).

### Inhibitory synapses do not link sigh bursts to the inspiratory rhythm

**Figure 4.**
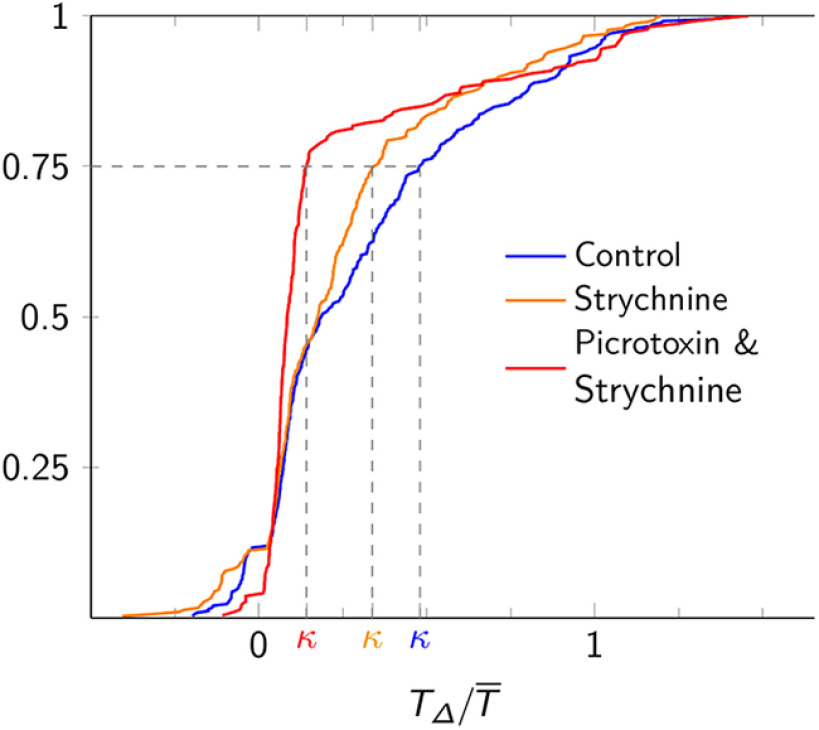
Cumulative probability histograms showing the normalized inspiratory-sigh interval ($T_{\Delta }/\bar{T}$) under control conditions (blue), 5 μm strychnine (orange), as well as 5 μm strychnine and 5 μm picrotoxin (red); κ marks the 75th percentile line for each distribution, distinguished by color. This analysis pools all sighs measured across all 13 slices.

To perturb the temporal coupling between the inspiratory and sigh rhythms we blocked glycinergic synaptic transmission using strychnine. The probability distribution of inspiratory-sigh intervals remained weighted toward the first half of the normalized cycle ($T_{\Delta }/\bar{T}$ < 0.5; Fig. 3*B*), suggesting that inspiratory-sigh coupling remained intact. In contrast, previous studies suggested that blocking glycinergic synapses eliminated the temporal relationship between sigh bursts and their preceding inspiratory bursts (Lieske et al., 2000; Chapuis et al., 2014), in which case the probability distribution in Figure 3*B* would be uniformly distributed between 0 and 1. Here, the probability of short inspiratory-sigh intervals ($T_{\Delta }/\bar{T}<0.5$) increased such that the mean inspiratory-sigh interval decreased from $T_{\Delta }/\bar{T}$ = 0.31 ± 0.34 in control (Fig. 3*A*, μ) to $T_{\Delta }/\bar{T}$ = 0.25 ± 0.30 in the presence of strychnine (Fig. 3*B*, μ). This trend is reflected in the significant leftward shift of the cumulative distribution function toward, but not below, an inspiratory-sigh interval of 0 from control to strychnine conditions (Kolmogorov–Smirnov, test statistic = 0.13, *p* = 0.011, *n* = 7 slices; Fig. 4). The left shift of the entire inspiratory-sigh interval distribution is further illustrated by the decrease of the 75th percentile score (Figs. 3, 4, κ) from $T_{\Delta }/\bar{T}$ = 0.48 in control to $T_{\Delta }/\bar{T}$ = 0.34 in strychnine.

Similarly, when we simultaneously blocked glycine and GABA_A_ receptors with a strychnine and picrotoxin cocktail, the sigh burst coupled more tightly with the preceding inspiratory burst than in control conditions, as shown by the accumulation of inspiratory-sigh intervals within the first two bins of the inspiratory-sigh interval histogram (Fig. 3*C*). The probability of short inspiratory-sigh intervals ($T_{\Delta }/\bar{T}<0.5$) increased from control to the strychnine and picrotoxin cocktail such that the average normalized inspiratory-sigh interval decreased from $T_{\Delta }/\bar{T}$ = 0.31 ± 0.34 in control (Fig. 3*A*, μ) to $T_{\Delta }/\bar{T}$ = 0.21 ± 0.32 in strychnine and picrotoxin (Fig. 3*C*, μ). The significant leftward shift of the cumulative distribution function toward, but not below, an inspiratory-sigh interval of 0 further demonstrates that when both glycine and GABA_A_ receptors were blocked, sigh bursts occurred earlier with respect to preceding inspiratory bursts than during control (Kolmogorov–Smirnov, test statistic = 0.31, *p* = 4.0E-12, *n* = 6 slices; Fig. 4). The 75th percentile score (Figs. 3, 4, κ) drops from an inspiratory-sigh interval of $T_{\Delta }/\bar{T}$ = 0.48 in control to $T_{\Delta }/\bar{T}$ = 0.14 in strychnine and picrotoxin. Whereas in control and strychnine conditions μ < κ (the mean of the distribution was within the 0–75th percentile range) in the combined presence of strychnine and picrotoxin κ < μ, which shows that the vast majority of inspiratory-sigh intervals (in 0–75th percentile range) are shorter than the mean.

Note that the large standard deviation associated with the average inspiratory-sigh intervals (0.31 ± 0.34 in control, 0.25 ± 0.30 in strychnine, and 0.21 ± 0.32 in strychnine and picrotoxin) reflects the inherent inspiratory-sigh interval variability as illustrated in Figure 3.

Our analyses (Figs. 3, 4) show the removal of chloride-mediated synaptic inhibition does not uncouple the sigh from its preceding inspiratory burst, but rather disinhibition strengthened the temporal coupling of inspiratory and sigh bursts.

### Inhibitory synapses do not influence post-sigh apnea

**Figure 5.**
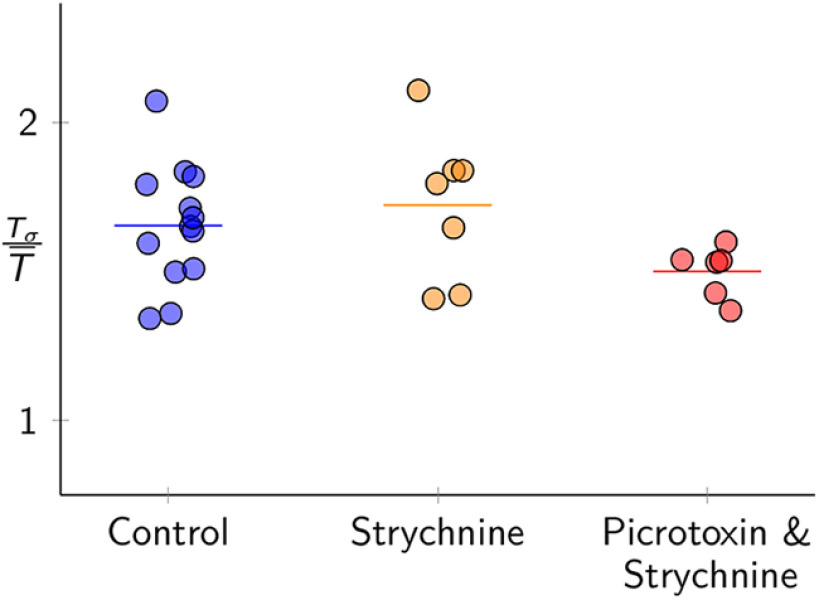
Average relative duration of the post-sigh apnea in control (blue), 5 μm strychnine (orange), as well as 5 μm strychnine and 5 μm picrotoxin (red). Each dot represents the average normalized post-sigh apnea for all sighs recorded in one slice. Horizontal bars represent the average of all slices in each condition.

We calculated the relative duration of the post-sigh apnea as the duration of the post-sigh interval ($T_{\sigma }$) divided by the average of the six preceding inspiratory cycle times ($\bar{T}$). We compared the relative duration of the post-sigh apnea ($T_{\Delta }/\bar{T}$) in control and after blocking either glycinergic transmission, or both glycinergic and ionotropic GABAergic transmission (Fig. 5). The relative duration of the post-sigh apnea measured 1.65 ± 0.20 in control, 1.72 ± 0.25 in strychnine, and 1.5 ± 0.09 in the strychnine-picrotoxin cocktail. These measurements are statistically indistinguishable (one-way ANOVA, *F*_(2,11)_ = 2.12, *p* = 0.14, *n* = 13 slices).

### Chloride-mediated synaptic transmission is inhibitory in the neonatal mouse preBötC

**Figure 6.**
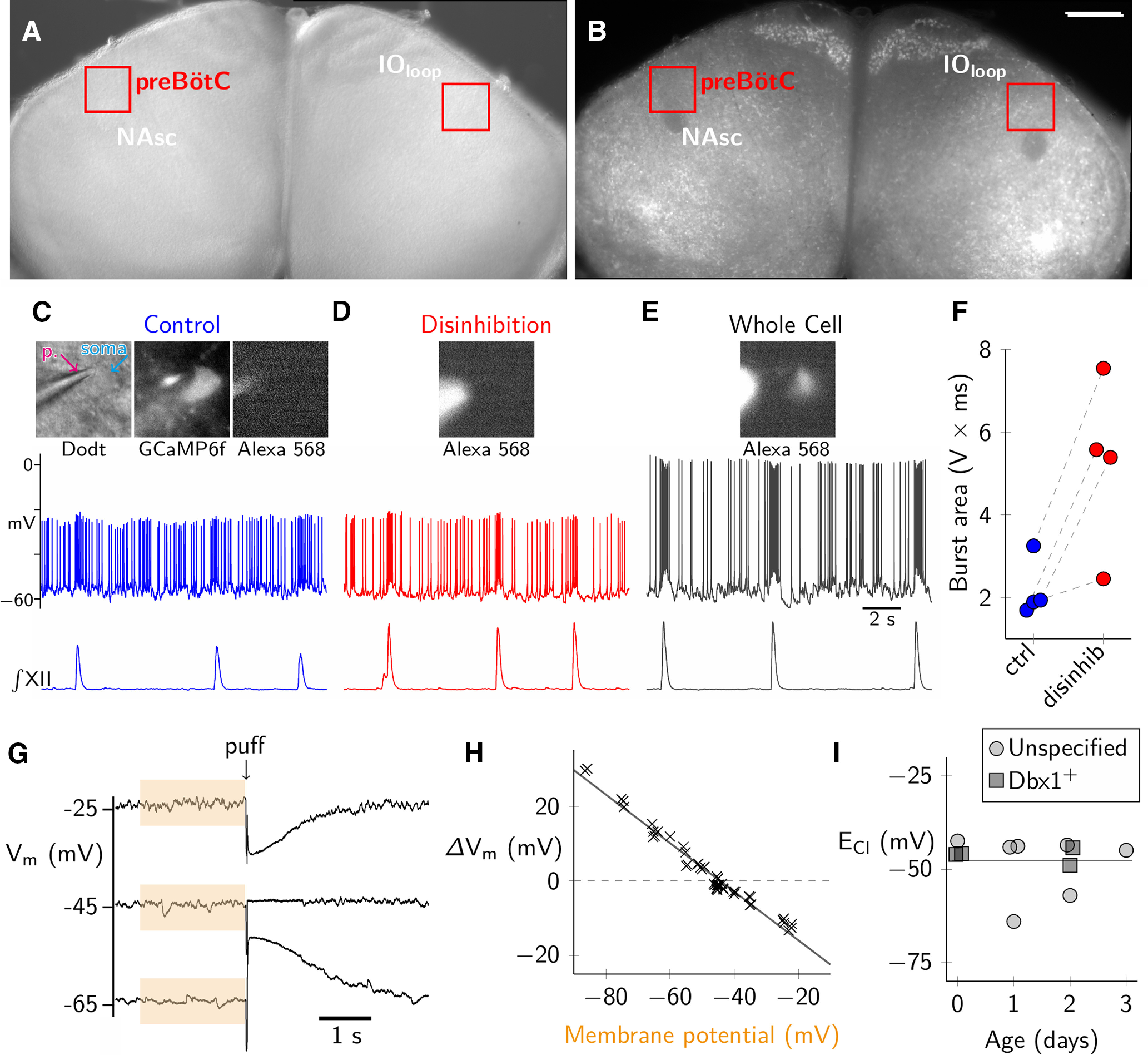
Chloride-mediated synapses are inhibitory in neonatal preBötC neurons. ***A***, ***B***, Image of Dbx1;Ai148 slice preparation under Dodt contrast (***A***) and fluorescence microscopy (***B***). preBötC (red box) colocalized with the NAsc and the principle loop of the inferior olive (IO_loop_). Scale bar for both ***A***, ***B***: 500 μm. ***C***, Voltage trajectory of a Dbx1 preBötC neuron during formation of a gramicidin perforated patch, firing in sync with XII nerve activity. Dodt image shows the patch pipette on the neuronal soma. The magenta arrow (labeled p.) points to the patch pipette, the cyan arrow (labeled soma) indicates the patched cell body. GCaMP6f image shows the neuron was *Dbx1* derived. The lack of fluorescence in the Alexa Fluor 568 image verifies the integrity of the perforated patch. ***D***, Membrane voltage trajectory of the same neuron during blockade of chloride-mediated synaptic inhibition via addition of 5 μm strychnine and 5 μm picrotoxin in the bath. Alexa Fluor 568 continues to verify the integrity of the perforated patch. ***E***, The same neuron in a whole-cell patch configuration shows Alexa Fluor 568 enters the soma from the patch pipette. The Dbx1 neuron is still rhythmically active and fires in sync with XII motor output. ***F***, Average integrated burst area during either control conditions (ctrl, blue) or during glycine and GABA_A_ receptor blockade (disinhib, red). ***G***, Determining E_Cl_. Voltage trajectory before and after puffer application of 150 μm glycine and 30 μm muscimol in TTX. The average baseline membrane potential before puffer application was calculated from the previous 2 s of recording (orange box). The puff-evoked change in membrane voltage (Δ V_m_) was the difference between baseline and the peak response. ***H***, Membrane potential changes in response to glycine and muscimol puffs plotted versus holding potential from a single representative experiment (same cell as ***G***). E_Cl_ (read from the *x*-axis coordinate where Δ V_m_ = 0 on the *y*-axis) is determined from linear regression (black line). ***I***, E_Cl_ from 11 neurons (four Dbx1 neurons from four Dbx1;Ai148 mouse slices, seven wild-type neurons) plotted as a function of postnatal age. Line at –48 mV indicates the average E_Cl_ across all neurons.

We recorded chloride-mediated synaptic potentials in preBötC neurons via gramicidin perforated patches during early postnatal development (P0–P3). These experiments employed wild-type mouse slices as well as slices from Dbx1;Ai148 mice that express a fluorescent Ca^2+^ reporter (GCaMP6f) in preBötC neurons derived from *Dbx1*-expressing progenitors (i.e., Dbx1 neurons), which comprise the core excitatory interneurons of the preBötC (Bouvier et al., 2010; Gray et al., 2010; Wang et al., 2014; Cui et al., 2016; Vann et al., 2016, 2018; Baertsch et al., 2018). Figure 6*A*,*B* shows the neuroanatomical structures from a Dbx1;Ai148 slice that collocate with the preBötC: the principal loop of the inferior olive (IO_loop_) and the NAsc. Figure 6*C* depicts the perforated patch configuration wherein GCaMP6f expression verifies that the neuron was *Dbx1* derived. Figure 6*C* also shows that Alexa Fluor 568 remains confined to the pipette solution during perforated patch recording conditions.

We applied strychnine and picrotoxin to the bath simultaneously while recording Dbx1 inspiratory neurons (*n* = 4 neurons in 4 slices). We monitored the integrated area of the membrane potential trajectory during inspiratory bursts while vigilantly monitoring the integrity of the perforated patch throughout the experiment using the absence of Alexa Fluor 568 in the soma as evidence of an intact patch (Fig. 6*C*,*D*). After blockade of chloride-mediated synapses, the average integrated area of inspiratory bursts increased from 2.2 ± 0.7 V × ms in control to 5.2 ± 2.1 V × ms (paired *t* test, *t* = 3.6, *p* = 0.037; Fig. 6*F*). The significant increase in burst area suggests that chloride-mediated synapses are functionally inhibitory during network bursts. As a final check, we switched from a perforated patch configuration to whole-cell and recorded the dialysis of Alexa Fluor 568 into the soma, concomitant with increased action potential amplitude, which confirmed the patch had not been breached during control and disinhibition data acquisition bouts (Fig. 6*D*,*E*).

Next, we measured E_Cl_ in 11 preBötC neurons (*n* = 7 unspecified preBötC neurons from wild-type slices, and *n* = 4 rhythmically active Dbx1 preBötC neurons, all from different slices). In the presence of TTX, pressure-pulse ejections of muscimol and glycine transiently perturbed the membrane potential, which reversed at or below −45 mV (Fig. 6*G–I*). E_Cl_ measured −48 ± 6 mV (mean ± SD, *n* = 11 preBötC neurons recorded in *n* = 10 slices). We observed no relationship between E_Cl_ and age (Fig. 6*I*), which indicates that E_Cl_ remains constant during P0–P3.

## Discussion

Eupnea and sigh rhythms are coordinated. The conventional understanding is that sigh breaths build off the crest of eupneic breaths, and then eupneic breaths resume after the post-sigh apnea, a pronounced delay that exceeds the gap between eupneic breaths normally. Here, we show that the longstanding conceptual framework for the eupnea-sigh relationship is oversimplified. We report previously unrecognized variability in the timing between eupneic and sigh breaths *in vivo*. The temporal coupling between eupnea and sigh rhythms, including its variability, may be attributable to dynamics within the preBötC because these same dynamics are mimicked in slice preparations that isolate the preBötC yet maintain both inspiratory and sigh rhythms.

The variability in the coordination of the inspiratory and sigh rhythms suggests that their coupling is weaker, relatively speaking, than previously appreciated. Sighs and sigh bursts *in vitro* with inspiratory-sigh intervals ≤0 s (where the sigh burst appeared coincident with, or before, the inspiratory burst) further reinforce this notion of flexibility in the relationship between a sigh burst and an associated inspiratory burst by showing that the inspiratory burst is not strictly necessary to trigger a sigh burst. Thus, a sigh can manifest independently, however, more often than not an inspiratory burst does trigger the sigh burst.

Could a sigh (or sigh burst *in vitro*) followed by a breath (or inspiratory burst *in vitro*) reflect a stand-alone sigh accompanied by a spurious artifact on the downslope of the sigh? Unlikely, we argue. In the case of animal movement during plethysmography, mice tend to move in bouts of activity lasting 1–10 s, which perturbs breathing over many consecutive cycles, and they rarely move in isolated 50- to 100-ms episodes that correspond to the duration of a putative eupneic breath whose peak follows a sigh (Fig. 2*A*). For preBötC field recordings, noise-driven network activity fluctuations with magnitudes large enough to be confused with inspiratory bursts are rare. Further, *in vivo* and *in vitro*, the observed breaths (bursts) that follow the sigh events match the preceding tidal volume (inspiratory burst amplitude), which argues that they are bona fide eupneic (inspiratory) events rather than recording artifacts. Nevertheless, if we accept the possibility that the trailing event is spurious, then those negative eupnea-sigh (or inspiratory-sigh) intervals would instead be closer to one, because the preceding eupneic or inspiratory event would have occurred one entire cycle away. This abnormally long eupnea-sigh (or inspiratory-sigh) interval would further support our conclusion that sighs follow eupneic or inspiratory events with variable intervals, and that a sigh burst has no obligatory link to the preceding inspiratory burst.

Environmental conditions like hypoxia (Janssen et al., 2000) or stress (Ramirez, 2014) modulate sigh frequency. Bombesin-like peptides delivered by parafacial inputs to the preBötC influence sigh frequency (Li et al., 2016). However, it is not clear whether modulation of sigh frequency would influence the eupnea-sigh coupling.

Sighs are 2–5× the tidal volume of eupneic breaths *in vivo*, whereas sigh bursts *in vitro* exceed inspiratory bursts by only 1- to 2-fold. Despite this disparity in sigh event magnitudes, the temporal relationship between the two rhythms measured *in vivo* or *in vitro* remains the same, as shown in the histograms from Figures 2, 3*A*. Those observations suggest that sigh magnitude does not influence eupnea-sigh (inspiratory-sigh) coupling. It is also remarkable that the sigh frequency remains constant (0.3–0.5 min^−1^) and the eupnea-sigh coupling pattern is conserved from intact adult mice to slices despite the fact that slices isolate the preBötC from all sensory feedback and some pattern-forming microcircuits. The robustness of the sigh frequency and the eupnea-sigh coupling suggests the sigh timing, and its coupling with eupnea, is largely attributable to microcircuit mechanisms within the preBötC.

We examined the mechanisms of coupling *in vitro*. Chloride-mediated synaptic inhibition is not responsible for the temporal coupling between inspiratory and sigh bursts. Rather, removing inhibition shortened the time between a sigh burst and the preceding inspiratory burst. This conclusion contradicts prior studies showing that blockade of glycinergic transmission decoupled sighs from their preceding inspiratory bursts and created free-running sigh burst rhythms that are independent from ongoing inspiratory rhythms (Lieske et al., 2000; Chapuis et al., 2014; Toporikova et al., 2015).

We propose that the discrepancy between those prior results and our present findings are attributable to the late embryonic reversal of the chloride electrochemical gradient. Before embryonic day (E)15.5 in mice, the dominant expression of cotransporter NKCC1 in brainstem and spinal cord neurons elevates intracellular chloride concentration (Ren and Greer, 2006; Delpy et al., 2008; Viemari et al., 2011) such that chloride currents are inward (i.e., excitatory) at the baseline membrane potential of rhythmically active preBötC interneurons. Perinatally NKCC1 expression decreases in parallel with increasing expression of the chloride symporter, KCC2, which lowers intracellular chloride concentration (Stil et al., 2009; Gackière and Vinay, 2015). In the mature state, dominant KCC2 expression ensures that the chloride equilibrium potential is more hyperpolarized than spike threshold as well as baseline membrane potential during rhythmic activity; chloride currents are outward and inhibitory.

Here, we show, in early postnatal mouse development (P0–P4) with elevated (9 mm) [K^+^]_o_ aCSF to boost slice excitability, that blocking chloride-mediated synapses leads to a larger depolarizing drive during inspiratory bursts. This suggests that GABA_A_ and glycinergic inputs are effectively inhibitory, diminishing the effects of excitatory synaptic drive during inspiration. E_Cl_ measured −48 mV during early postnatal development in the preBötC. This equilibrium potential is below spike threshold and approximates the level of baseline membrane potential during the interburst interval. Therefore, GABA_A_ and glycinergic inputs either shunt the membrane, rendering it less responsive to excitatory (depolarizing) drive, or hyperpolarize it directly during the preinspiratory phase or during the inspiratory burst itself when the membrane potential trajectory exceeds −48 mV. Thus, in our experimental context strychnine and picrotoxin truly block synaptic inhibition.

**Figure 7.**
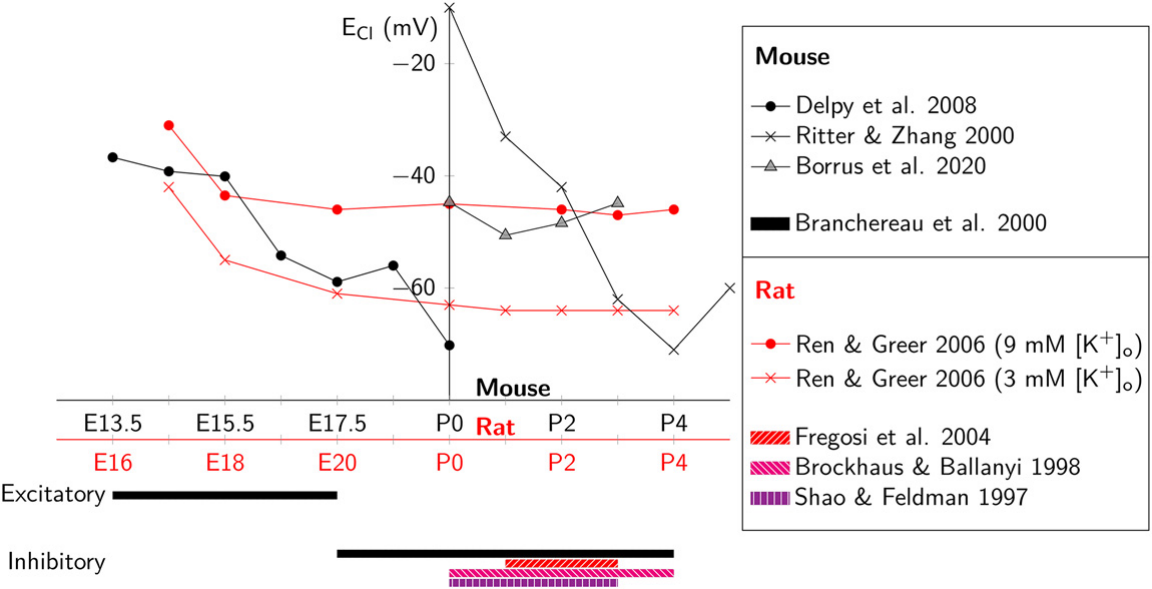
Meta-analysis of E_Cl_ during development in hindbrain and spinal cord of mice and rats. Red reflects rat data, black reflects mouse. The abscissa shows age (in days) centered at birth (P0). The ordinate shows E_Cl_ (in mV) as a function of age. Below the *x*-axis, we show binary classification of E_Cl_ as either above (excitatory) or below (inhibitory) baseline membrane potential as a function of developmental age. Our present data are labeled Borrus et al. (2020). We obtained ECl data from perinatal mice from: Branchereau et al. (2000), Delpy et al., 2008, Ritter and Zhang (2000), and data from the present manuscript, i.e., Borrus et al. (2020). We obtained ECl data from perinatal rats from: Brockhaus and Ballanyi (1998), Fregosi et al. (2004), Ren and Greer, 2006, as well as Shao and Feldman (1997).

We meta-analyzed the development of E_Cl_ in rodents in the context of our own E_Cl_ data (Fig. 7). Most studies agree that E_Cl_ switches from above to below baseline membrane potential (i.e., from excitatory to inhibitory) around birth, which is consistent with our measurement of E_Cl_ = –48 mV. However, there is substantial variability, which may be attributable to rodent species and strain differences, as well as choice of [K^+^]_o_ in the aCSF, which impacts the electrochemical forces that operate in transporters.

Whereas we studied postnatal (P0–P4) mice exclusively, Chapuis et al. (2014) and Toporikova et al. (2015) studied embryonic mice (E15.5–E18.5). In embryonic mouse slices bathed in elevated (8 mm) [K^+^]_o_ aCSF, E_Cl_ is more likely to be above spike threshold and consequently glycinergic synapses serve to depolarize and evoke action potentials in preBötC neurons (Ren and Greer, 2006; Delpy et al., 2008). Under these conditions, we infer that glutamatergic, glycinergic, and GABAergic synapses are all effectively excitatory and link sigh bursts to their preceding inspiratory bursts. When net excitatory drive is perturbed (such as by blocking chloride-mediated synapses) then inspiratory-sigh coupling weakens, and the sigh rhythm proceeds in a manner that is temporally independent of the inspiratory rhythm. Those results, in conjunction with our work showing disinhibition strengthens inspiratory-sigh coupling, leads us to our primary conclusion: that excitatory (not inhibitory) synaptic transmission links the inspiratory and sigh rhythms of the preBötC.

Development and chloride gradients might also explain the discrepancies between our results and Lieske et al. (2000), who also concluded that glycinergic synapses couple inspiratory and sigh rhythms. Those authors reported using mice aged zero to two weeks, a postnatal window that overlaps and extends beyond ours. We surmise that their sigh burst experiments were performed exclusively or predominantly using preparations from P0 mice with immature chloride gradients. As Figure 7 shows, Ritter and Zhang (2000) employed perforated-patch recordings and reported that E_Cl_ remained depolarizing and ostensibly excitatory as late as P3. Therefore, it is conceivable, even likely, that the same explanation holds for Lieske et al. (2000). Namely, chloride gradients favoring inward currents (with suprathreshold reversal potential) render glycine synapses ostensibly excitatory.

Chloride-mediated synaptic inhibition does not contribute to the post-sigh apnea. Instead, we suggest the post-sigh apnea is likely caused by activation of the intrinsic cellular mechanisms that help terminate inspiratory bursts, which are recruited to a greater degree during sigh events (compared with typical inspiratory cycles). These burst-terminating mechanisms include activity-dependent outward currents such as the electrogenic Na/K ATPase pump current, Na^+^-dependent K^+^ current, and ATP-dependent K^+^ current (Del Negro et al., 2009; Krey et al., 2010), as well as excitatory synaptic depression (Kottick and Del Negro, 2015; Guerrier et al., 2015). The magnitude of inspiratory burst-related depolarization directly evokes corresponding levels of post-burst hyperpolarization in preBötC neurons, from which the neurons must recover before generating the next inspiratory burst (Baertsch et al., 2018). The sigh burst in this context is an extreme version of that same mechanism: the increased magnitude and duration of the sigh event correspondingly evokes larger-than-average activity-dependent refractory (outward) currents and depresses excitatory synapses to a greater extent than during typical inspiratory bursts of lower magnitude and duration. The larger-than-average hyperpolarization (and depressed synapses) extends the duration of the interburst interval, thus creating the post-sigh apnea.

Here, we demonstrate that chloride-mediated synaptic inhibition plays no obligatory role in coupling the inspiratory and sigh rhythms in P0–P4 mice *in vitro*. Although sigh bursts are often closely preceded by inspiratory bursts, their temporal coordination is more variable than previously documented. We speculate that this principle may also apply to juvenile and adult stages because E_Cl_ is expected to remain below spike threshold and inhibitory; in fact, we expect it to descend lower than –48 mV during further maturation. A model of the preBötC core that generates inspiratory and sigh oscillations should emphasize the primacy of excitatory synaptic interactions, which probably extends to embryonic development when chloride-mediated synapses are functionally excitatory.

## Synthesis

Reviewing Editor: Douglas Bayliss, University of Virginia School of Medicine Decisions are customarily a result of the Reviewing Editor and the peer reviewers coming together and discussing their recommendations until a consensus is reached. When revisions are invited, a fact-based synthesis statement explaining their decision and outlining what is needed to prepare a revision will be listed below. The following reviewer(s) agreed to reveal their identity: Ralph Fregosi, Gregory Funk.

The reviewers were pleased with your revisions and your sincere and significant effort in addressing all their concerns. They also agree that this work convincingly demonstrates the novel observation that synaptic inhibition is not required for eupnea-sigh coupling, which occurs with variable timing.

Their comments are provided below.

Reviewer #2

Advances the Field

The observation that eupnea-sigh coupling is not dependent on synaptic inhibition is of interest to the field. Indeed, the data show that inhibition decreases eupnea-sigh coupling.

Comments to the Authors

Thanks for a rigorous revision of this interesting manuscript.

Reviewer #3

Advances the Field

The main contributions of the manuscript are that it demonstrates: 1. Significant variability in the temporal relation between onset on inspiratory and sigh-like events in vivo and in vitro 2. that inhibition is not necessary for the coupling of the two rhythms and that inhibition in fact, weakens the strength of the coupling, increasing the lag between inspiratory and sigh onset and increases the variability in this delay. The data convincingly support these conclusions.

Comments to the Authors

Authors have expended considerable effort in revising the manuscript a second time. I thank them for their attention to detail and careful consideration of points raised in review. They have addressed all my concerns. I think this will be a valuable and highly read paper.
